# Effect of α-Terpineol on Chicken Meat Quality during Refrigerated Conditions

**DOI:** 10.3390/foods10081855

**Published:** 2021-08-11

**Authors:** Khabat Noori Hussein, Barbara Csehi, Surányi József, Horváth Ferenc, Gabriella Kiskó, István Dalmadi, László Friedrich

**Affiliations:** 1 Department of Animal Production, College of Agricultural Engineering Sciences, University of Duhok, Zakho Street 38, Duhok 42001, Kurdistan Region, Iraq; 2 Department of Livestock Products and Food Preservation Technology, Institute of Food Science and Technology, Hungarian University of Agriculture and Life Sciences, Ménesi út 43-45, 1118 Budapest, Hungary; csehi.barbara@uni-mate.hu (B.C.); Suranyi.Jozsef@uni-mate.hu (S.J.); ferenc.horvath@spar.hu (H.F.); dalmadi.istvan@uni-mate.hu (I.D.); friedrich.laszlo.ferenc@uni-mate.hu (L.F.); 3 Department of Food Microbiology, Hygiene and Safety, Institute of Food Science and Technology, Hungarian University of Agriculture and Life Sciences, Somlói út 14-16, 1118 Budapest, Hungary; kisko.gabriella@uni-mate.hu

**Keywords:** bioactive compounds, physicochemical characteristics, antioxidant activity, antimicrobial activity, poultry meat

## Abstract

The present study was designed to evaluate the in vitro antimicrobial properties of nine bioactive compounds (BACs). Applying the disc paper and minimum inhibitory concentration (MIC) assays, we found that the BACs with the widest spectrum of in vitro antibacterial activity against the studied bacteria were carvacrol and α-terpineol (αTPN). Subsequently, αTPN was selected and applied at different concentrations into the fresh minced chicken meat. The meat was then vacuum packaged and stored for 14 days at 4 °C. Physicochemical properties, lipid oxidation (thiobarbituric acid reactive substances, TBARS), electronic-nose-based smell detection, and microbiological characteristics were monitored. At day 14, meat treated with higher concentrations of αTPN (MIC-2 and MIC-4) exhibited a significantly increased pH and lightness (L*), increased yellowness (b*), decreased redness (a*), caused a significant decrease in water holding capacity (WHC), and decreased lipid oxidation by keeping TBARS scores lower than the control. Although αTPN showed perceptibly of overlapped aroma profiles, the E-nose was able to distinguish the odor accumulation of αTPN between the different meat groups. During the 2-week storage period, αTPN, particularly MIC-4, showed 5.3 log CFU/g reduction in aerobic mesophilic counts, causing total inhibition to the *Pseudomonas* *lundessis*, *Listeria* *monocytogenes*, and *Salmonella* Typhimurium. These promising results highlight that αTPN is exploitable to improve the shelf life and enhance the safety of meat and meat products.

## 1. Introduction

The major quality attributes that correlate with the decreased shelf life of fresh chicken meat during refrigeration storage are physicochemical, microbial spoilage, lipid oxidation, and organoleptic changes, generating serious issues for both consumers and producers [1]. Due to the incidence of chemical and enzymatic activities as well as high-value nutrient composition, high water content, and moderate pH, meat is more highly perishable compared to a variety of foodstuffs [2]. It has been estimated that nearly 50% of the total meat produced globally is spoiled and wasted at the level of household consumption as a result of poor preservative techniques and facilities [3]. Despite the application of recent and advanced techniques, chemical preservatives, and cold chains in food preservation, it has been projected that microbial and chemical spoilage with other factors causes massive waste of approximately 1.3 billion tons/year (25%) of all food produced during post-harvest or post-slaughter [3,4]. If the meat and meat products are not preserved and handled properly it could be a common vehicle for foodborne diseases and compromises the nutritional quality, eventually leading to influence the meat physicochemical properties, product acceptance by consumers, and potential public health issues, causing food insecurity and economic concerns [5,6].

Lipid oxidation is the most common form of chemical, non-microbial cause of quality deterioration in meat during processing. As a result of the rapid depletion of endogenous antioxidants in meat after slaughter, oxidative damage can easily affect lipids and proteins [7]. Oxidation of lipid is a complex process, depending on the chemical composition of meat, light, and oxygen availability and storage temperature [8]. Lipid oxidation may develop (a) chemical spoilage; (b) degradation of pigments; (c) destruction of lipids, essential fatty acids, proteins, and fat-soluble vitamins and decrease of the energy content; (d) precipitate health hazards through the formation of carcinogenic substances (e.g., malondialdehyde—MDA), and developing potentially toxic substances in meat and food products (i.e., aldehydes, ketones, and alkanes); (e) contribute to drip losses; and (f) reduced shelf life, loss of nutritional value, and loss of functionality in meat products [9,10,11,12].

Moreover, microbial spoilage of meat during the supply chain is one of the major concerns causing quality defects and has the potential of causing food-borne illness and the development of unpleasant quality characteristics. Each year, about 600 million cases of food-borne illnesses and about 420,000 death-related cases are estimated globally by the World Health Organization (WHO) [13]. Major pathogenic bacteria include aerobic mesophilic count (AMC), *Listeria monocytogenes*, enterohemorrhagic *Escherichia coli* O157:H7, *Salmonella* spp., *Staphylococcus aureus*, *Bacillus cereus*, *Campylobacter* spp., *Clostridium perfringens*, and *Aspergillusniger* [2]. In chicken meat, the availability of AMCs is an indicator of the hygienic level, and usually in-ground chicken meat, the AMCs are high, which consequently increases the risk of microbiological spoilage disintegration. Moreover, in low-, middle-, and high-income countries, *S.* Typhimurium is associated with foodborne outbreaks such as the most dominant serovar globally [14]. *S.* Typhimurium has been associated with the consumption of undercooked meat or ground meat (poultry and beef), dairy products, and especially raw eggs. Salmonellosis has been known as one of the most common foodborne diseases globally, accounting for around 93.8 million foodborne illnesses and about 155,000 cases of deaths per year worldwide [15]. In the EU, *Salmonella* was found at 4.1% during the prevalence in 51,093 fresh broiler meat units [16]. Moreover, the *Pseudomonas* genus is one of the most significant distinct biologically groups of bacteria, containing more than 140 species, most of which are saprophytic [17]. Three species of *Pseudomonas* (*Pseudomonas fragi*, *Pseudomonas fluorescens*, and *Pseudomonas lundensis*) are mainly responsible for spoilage of variety of foodstuffs and colonize fresh meat and meat products (beef and chicken) [18,19,20]. It has been noticed that during processing, the scalding of poultry may destroy *Pseudomonas*, but it may increase the sensitivity of the carcass product to recontaminate at followed steps of processing [21]. On the other hand, *L. monocytogenes* is considered to be one of the important foodborne pathogens that causes foodborne disease called Listeriosis. The occurrence of *L. monocytogenes* in fresh broiler meat can varies from 0 to 64%, being produced during manufacture, ageing, transportation, and storage [22]. Since the discovery of *L. monocytogenes*, many control measures have been implemented, while an increase in listeriosis cases has been noticed. For instance, compared with 2012, the EU reported an 8.6% rise in listeriosis in 2013 [23] and an increase of 9.3% in 2016 compared to 2015, as recorded by EFSA [24]. These pathogens need to be controlled in the meat industry, and the best strategy to improve the safety of meat products throughout the stages of preharvest, postharvest, processing, storage, distribution, and consumption is providing adequate hygiene and the application of antimicrobial intervention technologies [25]. Concurrently with lipid oxidation, microbial spoilage leads to significant sensory abnormalities (texture and off-odor formations, causing discoloration and off flavors) in meat and meat products; therefore, exceptional protection is required to offer extended shelf life.

Various methods have been applied to limit the consequences of these detrimental factors affecting meat and meat products, including conventional thermal treatment and new strategies such as high hydrostatic pressure processing, ultrasound processing, MAP (modified atmosphere packaging) and vacuum packaging, irradiation, and pulsed electric fields (PEF) processing [26]. Moreover, using antioxidants/antimicrobials (for example, BACs) alone or in combination with technologies has been applied [27,28]. Synthetic additives are currently permitted for use in foods, but the trend of demands decreased due to the safety of synthetic chemicals and their potential toxicological and carcinogenic effects [29,30,31]. On the other hand, in recent years, the use of natural additives such as BACs as preservatives are gaining a wide interest and has attracted the attention of researchers. Many natural derivatives such as essential oils (EOs) and their BACs are documented and considered to be ‘Generally Recognized as Safe’ (GRAS) to be applied in different food systems and approved by the Food and Drug Administration (FDA), European Union, Council Directive No. 95/2/EC of 20 February 1995 regulation on food additives and European Commission (2002/113/EC, 2002) [32,33,34]. These BACs are receiving worthy attention for a number of a wide range of antimicrobial, flavoring, antioxidant, and organoleptic activities in preserving and improving the nutritional quality of food and meat products. Some of these BACs are of fruit and plant origins, such as carvacrol, thymol, allyl-isocyanate, eugenol, linalool, and piperine [34,35,36,37,38,39,40]. However, only low concentrations of BACs can be applied in meat preservation due to the serious flavor properties that may lead to change in the original flavor of the meat. 

α-Terpineol (*alpha*-*terpineo*l) is a volatile monoterpene relatively nontoxic alcohol. There are three isomers of terpineol, namely, alpha-, beta-, and gamma-terpineol, and the main isomer is αTPN ((*S*)-*p*-menth-1-en-8-ol), which comprises up to 30% of some EOs of different plant species and trees, such as eucalyptus globulus, pine oil, marjoram, oregano, thyme, Ravensara aromatica, cajuput oil, and Croton sonderianus [41]. αTPN is a relatively cheap and abundant aroma BACs that is widely used in cosmetics and household products [42]. The EOs that are rich in αTPN have been used widely in folk medicine for aromatherapy due to their anti-spasmodic, antinociceptive, and immunostimulant properties. Several studies have been conducted on αTPN to study its effects; for example, antimicrobial effects, anticonvulsant effects [43], its role as a potential anticancer agent [44], promising insecticidal activities [45], anti-inflammatory and anti-nociceptive central effects [46], and anti-hyperalgesic effects in an animal model [41]. To the best of our knowledge, no studies exist that deal with the preservative potential of αTPN in a food matrix, as is reported for some of its monoterpene counterparts. The overall objective of this study was to illustrate the application of αTPN in extending the shelf-life and improving the quality of fresh vacuumed minced chicken meat in refrigerated conditions.

## 2. Materials and Methods

### 2.1. Preparation of Raw Meat Samples and Experimental Design

Fresh chicken breast meat was obtained 24 h post-slaughtering from a local slaughterhouse and transported at 4 ± 0.5 °C to the laboratories. The meat was skin-off minced then homogenized using a meat grinder (BOSCH-Slovenia) and divided into treatment groups (the meat that contains BACs and control group). The samples were then placed in polyethylene bags, vacuum packaged, and stored at 4 ± 0.5 °C for up to 14 days. Experiments were conducted at room temperatures between 22 and 25 °C.

Disc diffusion and minimum inhibitory concentration (MIC) assays were applied to study the in vitro antimicrobial effect of BACs: p-cymene (99%), linalool (97%), camphor (96%), piperine (≥97%), γ-terpinene (97%), α-terpineol (>95%), α-pinene (98%), 1,8-cineole (98%), and carvacrol (98%) (SIGMA, Germany). On the basis of the in vitro antimicrobial activity and the MIC, we applied the BAC (αTPN) in MIC-1, MIC-2, and MIC-4 into minced chicken breast. For the meat treatment in MIC-1, the proportion of 5% of a mixture of 0.25 + 3.45 + 1.25 g of BAC + DW (distillated water) + ethanol, respectively, was used in 100 g meat (Table A1 in Appendix A). In MIC-2, the ratio of αTPN was twofold, and in MIC-4, the ratio of αTPN was fourfold. Samples were taken at different time intervals for different analyses on days 0, 3, 7, 10, and 14. Later, the physicochemical properties (pH, color, WHC), meat pigments, lipid oxidation (thiobarbituric acid reactive substances-TBARS), odor detection (E-nose-based smell detection), and microbiological properties (aerobic mesophilic counts—AMCs, *L. monocytogenes*, *S.* Typhimurium, and *P. lundensis*) of chicken meat were monitored.

### 2.2. Physicochemical Properties

#### 2.2.1. Measurement of pH

The pH value of meat samples was measured in different experiments (starting 24 h post-slaughter and immediately after mincing), and the readings were recorded in triplicate by immersing a pH electrode (Testo 206; Testo-AG, Titisee-Neustadt, Germany) into the minced samples.

#### 2.2.2. Color Measurement

The color values of minced meat were measured using CIELAB [47] scoring system. The following parameters were obtained: L* (lightness), a* redness (+*a*, red; −*a*, green), and b* yellowness (+*b*, yellow; −*b*, blue) by using a Konica Minolta CR-400 colorimeter (Konica Minolta Sensing Inc., Japan), making sure calibration was carried out before taking a reading from each treatment [48]. Results from L*, a*, and b* were recorded as the mean of five random readings, and from the measured values, relative colorfulness or chroma magnitude (C*) and hue angle (h*) were calculated as the following:

- Chroma: C* = [(a*)^2^ + (b*)^2^]^1/2^.
- Hue angle: h* = tan^−1^ (arctangent) (b*/a*).

#### 2.2.3. Measurement of Water Holding Capacity (WHC)

Measurement of water holding capacity was performed using the filter paper press technique. A sample (0.25–0.32 g) was placed on a filter paper (Whatman no. 10) set between 2 Plexiglas plates and pressed for 5 min by a 500 g weight. The filter paper was then placed in an oven for 10 min, followed by 5 min in a desiccator. WHC was calculated as the ratio of meat film area-to-total liquid outlined area [49]. Samples were measured in triplicate.

#### 2.2.4. Determination of Metmyoglobin, Deoxymyoglobin, and Oxymyoglobin Pigments

Meat pigment content was measured using the method applied by Utama et al. [50] with minor modifications, by which myoglobin was determined from absorbance measurements of the sarcoplasmic extract, dissolved in millimolar phosphate buffer (pH 6.8) from the reflex attenuance at 503, 525, 557, 572, 582, and 700 nm. Briefly, 2 g of sample was homogenized with 20 mL phosphate buffer using a homogenizer (Digital Ultra-Turrax, Staufen, Germany) at 10,000 rpm for 20 s. The homogenate was centrifuged at 5500× *g* for 30 min. The supernatant was filtered through filter paper then used for measuring the absorbance. The presented values are the mean of triplicate measurements per sample. The relative proportions (%) of each myoglobin form: oxymyoglobin (OxyMb), metmyoglobin (MetMb), and deoxymyoglobin (DeoMb) were calculated according to the method updated by Tang et al. [51]; the calculation was performed as below:

% MetMb = (−0.159𝑅1 − 0.085𝑅2 + 1.262𝑅3 − 0.520) ∗ 100
% DeoMb = (−0.543𝑅1 + 1.594𝑅2 + 0.552𝑅3 − 1.329) ∗ 100
% OxyMb = (0.722𝑅1 − 1.432𝑅2 − 1.659𝑅3 + 2.599) ∗ 100
*𝑅1 = 𝐴582/𝐴557, 𝑅2 = 𝐴557/𝐴525, and 𝑅3 = 𝐴503/𝐴525

### 2.3. Determinations of Thiobarbituric Acid-Reactive Substances (TBARS)

Lipid oxidation was determined by analyzing the thiobarbituric acid reactive substances (TBARs) index according to Dias et al. [48]. Five gram portions of chicken breast meat samples were combined with 20 mL of 5% trichloroacetic acid (TCA) (SIGMA, Darmstadt, Germany), and to prevent oxidation during the preparation, 0.5 mL of 0.15% BHT antioxidant (2,6-ditert- butyl-4-methylphenol) (SIGMA, Darmstadt, Germany) was used, and the samples were homogenized (Digital Ultra-Turrax Disperser, Germany) for 2 min. The homogenates were then centrifuged (5000× *g* for 10 min), the supernatant was filtered through filter paper into 25 mL volumetric flasks, and 5% TCA was added to reach a final volume of 25 mL. Two milliliters of filtrate were combined with 2 mL of 0.08% *w/v* TBA (0.02 M) (SIGMA, Steinheim, Germany) reagent, and the tubes were then sealed and placed in a water bath (95 °C) for 30 min. After cooling, the samples were vortexed, and absorbance of the resulting solution was measured at 532 nm using a Spectrophotometer (U-2900 Hitachi Ltd., Tokyo, Japan) against a blank containing all of the reagents except the sample, and the TBAR values were expressed as milligram of malondialdehyde (MDA equivalent) per kilogram sample [52,53].

### 2.4. Microbiological Properties

#### 2.4.1. In Vitro Anti-Microbial Activity of BACs

Six bacterial strains, three Gram-positive (G+veB) (*Listeria monocytogenes* CCM 4699, *Staphylococcus aureus* ATCC 6538, and *Bacillus cereus* T1) and three Gram-negative (G-veB) (*Escherichia coli* O157:H7 BO1909, *Salmonella* Typhimurium B1310, and *Pseudomonas lundensis* CCP5), were used as target bacteria in in vitro antimicrobial tests that were obtained from the Department of Food Microbiology, Hygiene and Safety, Institute of Food Science and Technology. Each strain was grown on a plate containing 25 mL sterile Tryptic-Soy agar (TSA) (Biokar Diagnostics BK046HA) at 37 °C for 24 h (except *P. lundensis*, which was incubated at 30 °C for 24 h).

#### 2.4.2. Disc Diffusion Assay

The test was performed in a sterile Petri dish (90 mm diameter) containing 20 mL TSA. Plates were inoculated with 1 mL of the target bacterium (approximately 10^6^ CFU/mL, set by measuring OD) on the agar surface. After a few minutes, the plates were sloped, and the access inoculum was removed by pipetting. Then, a sterile 5 mm diameter disc-shaped filter paper (Whatman no. 1, ≥10.5 cm in diameter) was placed on the middle of the agar surface, and 4 μL of BACs (p-cymene, linalool, camphor, piperine, γ-terpinene, αTPN, α-pinene, 1,8-cineole, and carvacrol) were applied on it (undiluted or diluted BACs in ethanol (96%)). For control, 4 μL of a sterile solution of ethanol (96%) was used. Each plate was sealed well with parafilm to prevent evaporation from the samples as well as the loss of volatile components of BACs [54]. Plates were incubated for 24, 48, and 72 h at either 30 or 37 °C according to the growth temperature requirement of the bacteria. The inhibition zone (mm) (colony-free perimeter) around the disc (starting from the edge of the disc) was measured using a Digital Vernier Caliper (Workzone-Caliper, Tokyo, Japan). The experiments were repeated in triplicate for all the tested strains.

#### 2.4.3. Minimal Inhibition Concentration (MIC)—Micro-Dilution Method

The MIC was determined using microdilution of tryptic soy broth (TSB) in 96-well plates. The stock solution was prepared by diluting 200 μL/mL of BACs in absolute ethanol in order to enhance their solubility. Non-liquid BACs were also diluted in ethanol (camphor 200 mg/200, piperine 12 mg/200 μL).

The MIC was determined using the resazurin microtiter plate-based antibacterial assay as described by Semeniuc et al. [55] with minor modifications. A total of 100 µL of TSB with 100 µL of sterile DW was pipetted into each well, and 100 µL of appropriately diluted BACs (from the stock solution) were placed in the well of the first column. Micro dilution and mixing did via a pipette 2–3 times for homogenization, then serial 11-fold dilutions were performed by transferring 50 μL into the right well and continued to the last well of the plate row. Then, 30 μL of the bacterial cell suspensions (10^6^ cells/mL) were pipetted into the appropriate well. Ethanol was also used as a control. After incubation, 10 μL aqueous mixture of resazurin (see later) was pipetted to each well. The final volume in each well was 290 µL. Microbial growth was indicated by color change. If the color stayed blue, it means there was no growth. If the color changed to pink, that means there was growth. Moreover, the intensity of the color depended on the amount of growth. Microplates were incubated at 37 °C for 24 h (except *P. lundensis*, which was incubated at 30 °C). The concentration that completely inhibited bacterial growth was designated as MIC. Three replicates were run for each BAC.

Resazurin solution made by diluting 0.025 g of resazurin sodium salt in 1 mL sterile DW and added to a pre-weighed medium of 8 mL TSB (double tryptic soy broth) and distributed in Eppendorf tubes (in each tube, 900 μL). Then, 0.014 g of menadione was diluted in 1 mL of DMSO, separately, and following this, menadione was added to previously made resazurin solutions to the stock solution. The stock solution was stored in a freezer at −20 °C. A total of 10 μL of this stock solution was added to each well of the plate after incubation.

### 2.5. Determination of Aerobic Mesophilic Counts (AMCs), Pseudomonas lundensis, Listeria monocytogenes, and Salmonella Typhimurium in Meat

#### 2.5.1. Preparation of Bacterial Strains and Inocula

The microbiological challenge test was carried out as a useful method in determining the potential shelf life of refrigerated meat. Both G+veB (*L. monocytogenes* CCM 4699) and G-veB (*S.* Typhimurium B1310 and *P. lundensis* CCP5) were used as target bacteria in antimicrobial tests. Cultures were streaked on TSA plates and incubated for 24 h at 37 °C (except *P. lundensis* incubated at 30 °C). The inocula of the test organisms were prepared by transferring a single colony from culture plates into 100 mL TSB and culturing at 37 °C for 24 h. These cultures were further used for testing the antimicrobial activities and for the inoculation of chicken breasts.

#### 2.5.2. Bacterial Inoculation on Chicken Meat

The meat samples (approximately 10 g/bag) were then inoculated with 10 µL mixtures of *L. monocytogenes, S.* Typhimurium, and *P. lundensis* bacterial solution from 300 mL TSB (100 mL/strain) with an initial cell count of 6–7 Log CFU/mL for each inoculated bacterium (inoculated control and treated samples). The meat was then vacuum packaged. This meat (10 g/bag) was stored at 4 °C until the day of measurement.

#### 2.5.3. Microbial Enumeration

Each sample (10 g/bag) was suspended aseptically with 40 mL of sterile saline solution, and the samples were homogenized in a sterile filter containing Stomacher bag for 2 min (Inter-science, Saint-Nom-la-Bretèche, France). Decimal serial dilutions were performed with sterile 0.1% peptone water. The microbial populations were quantified by spreading 100 µL from the homogenized meat bag and plated using the following media: xylose lysine deoxycholate agar (XLD) (SIGMA, Darmstadt, Germany) for *Salmonella*, PALCAM (polymyxin acriflavine lithium chloride ceftazidime aesculin mannitol) (SIGMA, Darmstadt, Germany) for *L. monocytogenes*, cetrimide agar (SIGMA, Germany) for *Pseudomonas*, and tryptone glucose extract (TGE) for AMCs. For preparing the TGE, to one liter of sterile DW we added 0.5% peptone, 0.1% glucose, 0.25% yeast extract, and 1.5% bacteriological agar [38]. *Listeria* and *Salmonella* plates were thoroughly shacked before solidification and then incubated for 24 h at 37 °C, and AMCs and *Pseudomonas* were incubated at 30 °C before enumeration. The results are expressed as the logarithms of colony-forming units per gram of sample (log CFU/g).

### 2.6. Electronic Nose Analysis

Electronic nose determinations were performed with an NST 3320 instrument (Applied Sensor Technologies, Linköping, Sweden) as described by Friedrich et al. [56]. This instrument has a built-in headspace sampler for 12 samples, a detector unit containing 23 different sensors, and software for collecting and processing data from the sensors. NST 3320 is equipped with 10 MOSFET (metal-oxide semiconductor field-effect transistor) sensors, 12 MOS (metal oxide semiconductor) sensors, and a humidity sensor for measuring relative humidity. The MOSFET sensors are based on a change in electrostatic potential. Eight gram meat samples (three replicates each) were filled to special glass vials that were closed by a septum. The standby temperature, at which the samples were kept until their incubation phase started, was 20 °C. Before analysis, the samples were equilibrated at 60 °C for 30 min (incubation phase). The total cycle time per sample was 430 s. The difference of sensor signals between the baseline and the signal value at the end of the sampling time was used for multivariate statistical analysis as a sensor response.

### 2.7. Statistical Analysis

The experimental data were analyzed using SPSS (Version 23.0, SPSS Inc., Chicago, IL, USA). The data were subjected to analysis of variance (ANOVA) and general linear model (GLM), and then the level of significance was established using the Tukey test at (*p* < 0.05). In physiochemical and lipid oxidation analysis, the mean data ± standard deviation has been presented. Microbiological data were converted to Log CFU/g. In the case of E-nose measurements, canonical discrimination analysis (CDA) was applied to distinguish between different meat groups.

## 3. Results

### 3.1. Evaluation of the In Vitro Antimicrobial Activity of BACs

#### 3.1.1. Using Disc Diffusion Assay

The antibacterial activity of the individual BACs using the filter paper disc diffusion method is summarized in Table 1. The components with the widest spectrum of antibacterial activity against the studied bacteria were found to be carvacrol, followed by linalool, αTPN, α-pinene, 1,8-cineole, γ-terpinene, camphor, and *p*-cymene. After 24 h incubation, carvacrol showed 5.19 ± 0.02, 20.14 ± 0.73, 16.70 ± 0.29, 17.27 ± 1.00, 15.15 ± 0.27, and 17.60 ± 0.39 mm inhibition zone for *P.* *lundensis*, *E. coli*, *S. aureus*, *L. monocytogenes*, *S.* Typhimurium, and *B. cereus*, respectively (Table 1). Moreover, linalool and αTPN showed a zone of inhibition against all the studied strains. On the other hand, α-pinene did not show inhibitory activity against *L. monocytogenes* and *B. cereus*. Additionally, camphor only was active against *E. coli* and *p*-cymene against *S. aureus*, and piperine did not exhibit antimicrobial activity using the disc method.

#### 3.1.2. Using the MIC Method

The antibacterial effects of various BACs against six foodborne and spoilage bacteria in a liquid phase (MIC values) using micro-dilution are presented in Table 2. Carvacrol showed the best activity among all the BACs, followed by αTPN and linalool. In contrast, α-pinene and γ-terpinene were found to be less active using the MIC assay. Among the BACs, αTPN was chosen as one of the most effective BACs in the liquid phase against *P. lundensis*, *E. coli*, *S. aureus*, *L. monocytogenes*, *S.* Typhimurium, and *B. cereus* due to its overall lower MIC values against these pathogenic bacteria as compared to the other BACs, except linalool and carvacrol, which have been studied in our previous work [36,37].

### 3.2. Physicochemical Properties

#### 3.2.1. pH of Meat

The result of the physicochemical properties of chicken meat treated with αTPN is listed in Table 3. Different concentrations of αTPN were able to alter the pH values of chicken meat during 14-day storage. At the end of the storage, the pH value of treated meat was increased significantly except for αTPN-MIC-4, which remained at high values 6.09 to 6.12 at days 0 and 14, respectively, compared to a significant decline in pH of control samples 6.02 and 6.01 at the same days (*p* < 0.05). Regarding the concentration of αTPN, significant differences were observed within groups containing αTPN and compared to untreated meat (*p* < 0.05).

#### 3.2.2. Color Values

The color of chicken meat showed significant changes (except redness values) during the 14-day storage period (Table 3). The increase rates were observed in all meat samples; however, the trend was most abundant in intensifying drifts in the lightness of the sample, which contained a high level of αTPN (MIC-2 and MIC-4) compared to control and MIC-1. At the end of the storage period, no significant differences were found with the addition of a low level of αTPN (MIC-1) compared to control, and it was effective in keeping the L* values close to the initial L* values, whereas the significant difference was noticed in MIC-2 and MIC-4 compared to untreated meat. The a* values in meat containing higher-level αTPN decreased at the end of storage compared to an increasing trend in control, but no significant changes were noticed. However, this decrease in the a* value was less in the meat treated with αTPN-MIC-1, which was close to the initial a* values at the beginning of storage. The b* value of the control and αTPN-MIC-1 decreased at day 14 of storage, unlike the reverse trend with a significant difference was observed in meat treated with αTPN MIC-2 and MIC-4. Regarding the concentration of αTPN, the meat containing MIC-2 and MIC-4 resulted in significantly higher b* compared to MIC-1 and control (Table 3). Similar to yellowness, increasing trends of color intensity (C*) were detected at day 14 in samples containing a higher rate of αTPN compared to a slight decrease with no significant rate in MIC-1 and control. The C* values in MIC-2 and MIC-4 were 13.18 ± 0.68, and 15.40 ± 1.57, respectively, on the first day and increased to 15.08 ± 1.16 and 16.71 ± 1.06, respectively at day 14, while for control and MIC-1, they were 12.87 ± 0.68 and 12.67 ± 1.18, respectively, at the first day and decreased to 11.81 ± 0.33 and 12.63 ± 0.57, respectively, at day 14 of storage. On the other hand, throughout the storage period, the steadiness was detected in the hue values (h*), despite a slight decrease in control and a slight rise in treated meat with no significant difference. However, regarding the concentrations of BACs, significant differences were noticed only on days 10 and 14, and only between treated meat and control. To the best of our knowledge, no studies have seen that dealing with the color changes and the preservative potential of αTPN in meat.

#### 3.2.3. Water Holding Capacity

During the 14-day storage period, different levels BACs, particularly αTPN MIC-2 and αTPN MIC-4, were able to show a significant effect on decreasing WHC (Table 3). On the other hand, no significant variation was witnessed in MIC-1 and control at end of the storage. Simultaneously the variation was noticed between the treated meat with a higher concentration of BAC compared to the control.

#### 3.2.4. Meat Pigments (Metmyoglobin, Deoxymyoglobin, and Oxymyoglobin)

**Figure 1:**
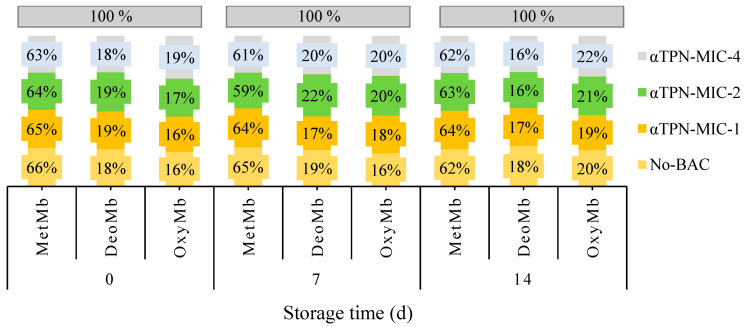
The influence of αTPN on fresh chicken meat pigments (metmyoglobin (MetMb), deoxymyoglobin (DeoMb), and oxymyoglobin (OxyMb)) stored up to 14 days at 4 °C.

The results from the αTPN on the profile of Mb pigments in chicken meat are presented in Figure 1. The initial percentages of MetMb in control, MIC-1, MIC-2, and MIC-4 were 66, 65, 64, and 63% and decreased to 62, 64, 63, and 62, respectively, on day 14. Besides the initial percentage of DeoMb in control, αTPN MIC-1, MIC-2, and MIC-4 were 18, 19, 19, and 18% and became 18, 17, 16, and 16%, respectively, at the end of storage. On the other hand, the percentage of OxyMb increased during the storage period. In control samples, no considerable changes were noticed in DeoMb, while decreased rate was found in MetMB and increased rate was observed in OxyMb.

### 3.3. Thiobarbituric Acid-Reactive Substances (TBARS)

**Figure 2:**
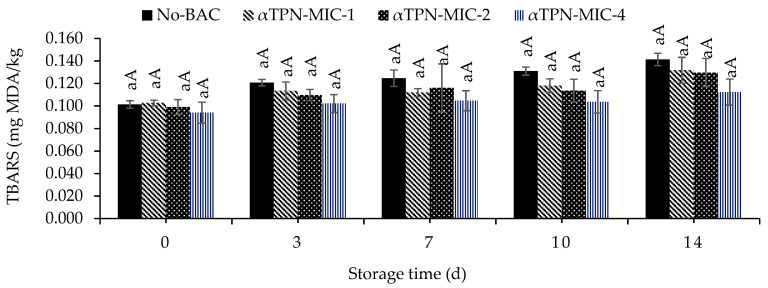
Effect of different concentrations of αTPN on TBARS values of fresh chicken meat stored up to 14 days at 4 °C. ^a^—letter with superscript is significantly regarding the days of storage; ^A^—letter with superscript is significantly regarding the concentrations of BACs (*p* < 0.05).

In this study, at the end of storage, the control group showed higher TBARS values compared to the rest of the samples, an increasing trend was noticed in the meat containing αTPN, whereas comparing to control, it showed a reduction in TBARS values with no significant variation (Figure 2). The reduction was more pronounced in meat treated with MIC-4, which had a positive effect in inhibiting oxidation and resulted in controlling the TBARS value from 0.094 on the first day of storage to 0.112 mg MDA/kg at day 14 compared to the control, which was increased from 0.101 to 0.141 mg MDA/kg. This result indicates the antioxidant activity of αTPN by keeping TBARS scores lower than 2 mg MDA/kg in chicken meat.

### 3.4. Microbiological Characteristics of Chicken Meat

**Figure 3:**
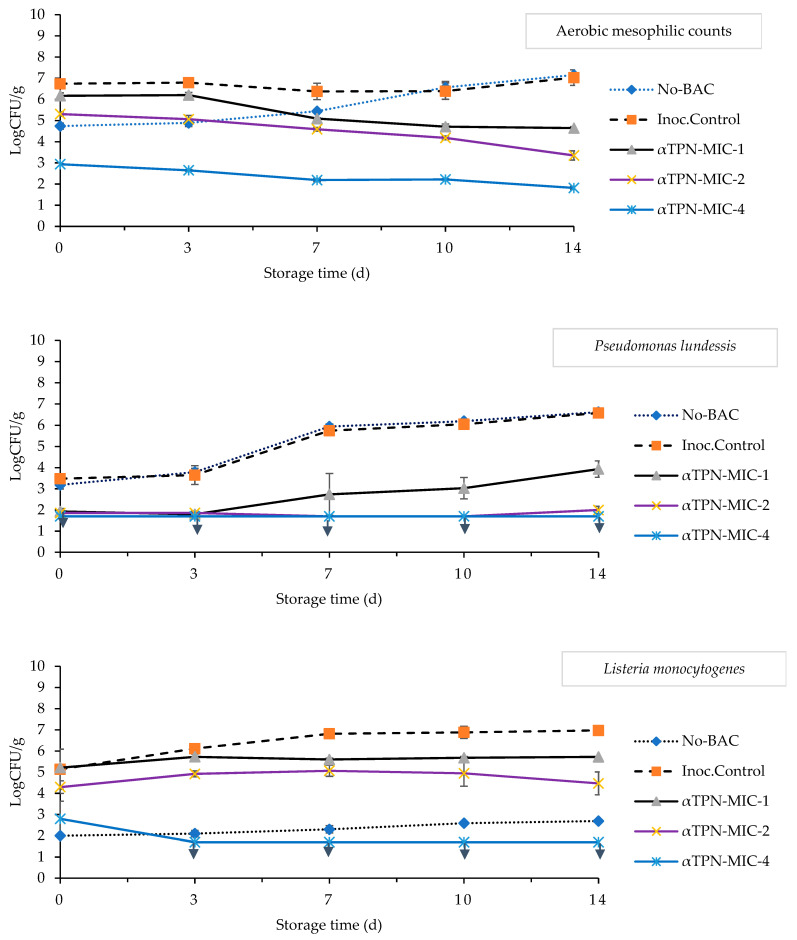

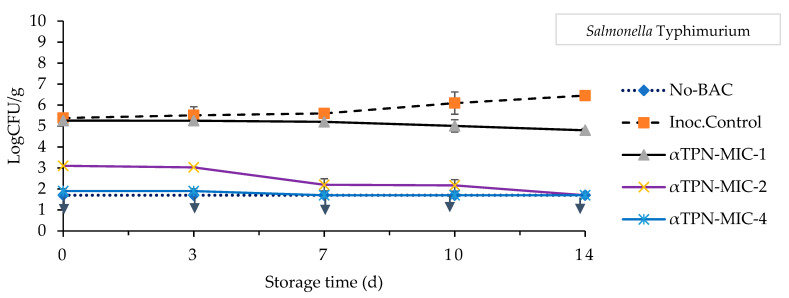
Effect of different concentrations of αTPN on aerobic mesophilic counts (AMCs), *Pseudomonas lundessis*, *Listeria monocytogenes*, and *Salmonella* Typhimurium in chicken meat stored up to 14 days at 4 °C. Arrow line (↓) represents the lower detection limit.

The results from the antimicrobial efficacy of αTPN against aerobic mesophilic counts (AMCs), *L. monocytogenes*, *S.* Typhimurium, and *P. lundessis* in chicken meat are presented in Figure 3. The initial AMCs population (day 0) in control was 4.74 log CFU/g as a characteristic of acceptable quality chicken meat. The highest count was in control and inoculated control on day 14, which were 7.15 and 7.03 log CFU/g, respectively. It is clear that αTPN had a profoundly higher effect on the inhibition of AMCs, because as the concentration of αTPN increased, the surviving count of AMCs decreased; αTPN MIC-1, MIC-2, and MIC-4 caused 2.5, 3.8, and 5.3 log CFU/g reduction in AMCs, respectively, for 2-week storage (Figure 3).

During storage, the cell count of *P. lundessis* increased in all meat groups except for the sample that contains αTPN MIC-4, which did not exhibit growth during the storage (Figure 3). The highest cell count of *P. lundessis* was seen in both controls and inoculated control, both showing 6.6 log CFU/g at day 14. On the other hand, meat treated with MIC-1 and MIC-2 the 1.9 log CFU/g cell count of *P. lundessis* was detected at day 0 which increased gradually to 3.9 and 2.0 log CFU/g on day 14 in meat that contained MIC-1 and MIC-2, respectively. On the other hand, cell counts of *L. monocytogenes* in chicken meat samples slightly decreased in meat containing αTPN, and even higher decrease level was noticed for samples treated with αTPN MIC-4, which reduced the cell count of *L. monocytogenes* from 2.8 to 1.7 log CFU/g at day 14. In control samples, *L. monocytogenes* started to show growth at day 7, while the inoculated control showed the highest counts, increasing from 5.1 to 6.9 log CFU/g, and meat treated with αTPN MIC-1 remained relatively stable throughout the storage. The counts of *S.* Typhimurium were not detected in control meat. The highest growth was observed in inoculated control, which reached 6.5 log CFU/g at day 14 (Figure 3). However, the cell counts of *S.* Typhimurium were decreased in meat treated with αTPN, and the high concentration MIC-2 and MIC-4 caused total inhibition to the pathogen at the end of the storage. Additionally, MIC-1 of αTPN reduced the cell count from 5.3 to 4.8 log CFU/g at day 14.

### 3.5. Electronic Nose

**Figure 4:**
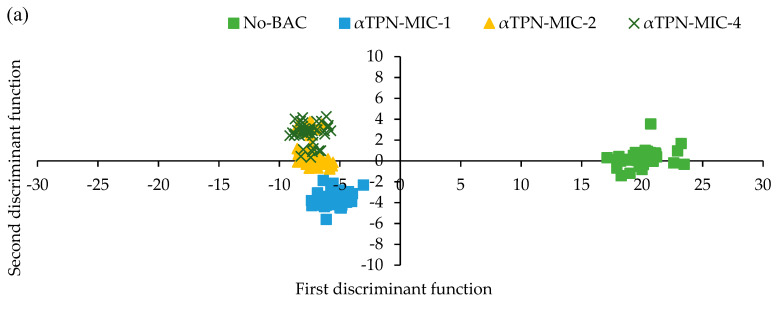

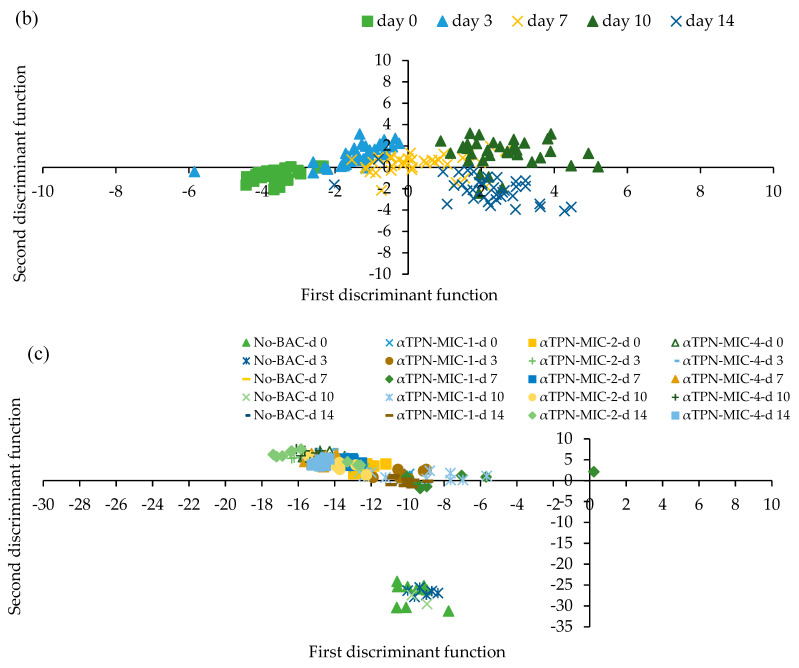
Efficacy of different concentrations of αTPN on smell detection by E-nose in chicken meat stored up to 14 days at 4 °C. Canonical discriminant analysis score plot of (**a**) the separation based on the concentration of BACs, (**b**) the separation based on storage days, and (**c**) the separation based on storage days and concentration of BACs.

The E-nose was applied to examine the group separation of meat samples treated with/without αTPN (Figure 4a–c). Correct distinguishing between untreated and treated meat on the basis of the concentrations of BAC and storage time was observed using E-nose. Comparing the different concentrations of αTPN, we found that the treated groups exhibited entirely different directions compared to untreated meat, and overlapping was only seen between MIC-2 and MIC-4 (Figure 4a). Additionally, different concentrations of αTPN showed the separation of treated meat on day 0 and day 14 of storage with a clear tendency toward second discriminant function, whereas comparison of the concentration of αTPN and the storage time the E-nose showed overlapping between the treated meat with clear pattern recognition and a tendency toward second discriminant function compared to control that remained at the first discriminant function.

## 4. Discussion

Consumers are increasingly concerned about synthetic preservatives used in food due to their potential toxicological and carcinogenic effects [31,57]. To obtain a clear illustration of the application of αTPN as a natural preservative in foodstuff, we included several aspects in this study. Mainly the physicochemical properties, lipid oxidation, odor detection (E-nose based smell detection), and in vitro and in meat microbiological properties (aerobic mesophilic counts, *L. monocytogenes*, *S.* Typhimurium, and *P. lundensis*) of chicken meat were examined.

### 4.1. Evaluation of the In Vitro Antimicrobial Activity of BACs

In this study, the disc paper and MIC method showed that carvacrol had the highest antibacterial activity against the studied bacteria, followed by αTPN. Furthermore, some BACs are well documented in the literature for their antimicrobial activity. Kim et al. [58] studied the antimicrobial properties of some BACs against four G-veB bacteria (*E. coli*, *E. coli* 0157:H7, *S.* Typhimurium, and *Vibrio vulnificus*) and one G+veB bacterium (*L. monocytogenes*). Using disk diffusion method, they ranked BACs effect against *E. coli* as linalool > eugenol > terpineol > carvacrol and against *S.* Typhimurium as linalool > eugenol > terpineol > carvacrol. In accordance with our findings, they found that carvacrol (minimum bacterial concentration—MBC 250 μg/mL) was most active against all the tested strains. However, the findings of Kim et al. [58] were not in agreement with ours as they found that αTPN and linalool were least potent against the studied strain. Guimarães et al. [59] observed that thymol, carvacrol, and eugenol presented strong antimicrobial action against *B. cereus*, *S.* Typhimurium, *E. coli*, and *S. aureus*, while *m*-Cymene, (±)-linalool, camphor, *trans*-Geraniol, terpineol, (±)-citronellal, (+)-borneol, and *R*-(+)-limonene demonstrated the least action and BACs such as *p*-cymene, (+)-α-pinene, ƴ-terpinene, (−)-α-bisabolol, and eucalyptol showed no activity against these strains. In vitro study on the antimicrobial activity of αTPN reported that due to the presence of OH, this BACs interacts with intracellular components and causes a change in the permeability of the outer membrane and a change the function of the cell membrane, leading to the leakage of intracellular materials [60]. López et al. [61] found that thymol and carvacrol showed significant antimicrobial activity against yeast (*Candida albicans*), molds (*Aspergillus flavus*), and G+veB (*L. monocytogenes*), but linalool was active against *Salmonella* choleraesuis and *Candida albicans*, while other BACs, camphor, esragol, 1.8-cineole, *p*-Cymene, and limonene, did not show any inhibitory activity against these microorganisms. Poor solubility in aqueous may reduce the antimicrobial activity of some BACs. Zengin and Baysal [60] also determined the MIC values of αTPN, which was 0.6% for *E. coli* O157:H7, *S. liquefaciens*, *C. divergens*, and *L. innocua,* while 0.7% of αTPN was needed to inhibit *S.* *aureus* and *S.* Typhimurium. They also observed that αTPN and linalool showed synergistic effects and αTPN/eucalyptol showed additive effects against *S*. Typhimurium, *E. coli* O157:H7, and *S.* *aureus*. Li et al. [62] demonstrated that the MIC and MBC values of αTPN against *E. coli* (CMCC (B)) were 0.78 μL/mL. They found that αTPN exhibited decreased cell size and irregular cell shape, cell wall, and ruptured cell membrane. It means that αTPN might inhibit the growth of *E. coli* by killing bacteria directly.

### 4.2. Effect of αTPN on the Physicochemical Properties of Chicken Meat

The physicochemical characteristics are regarded as one of the essential factors for consumers in determining meat quality and meat freshness [5]. Instrumental color measurement systems such as CIELAB scoring system are used as indicators of meat quality and a predictor of the preferred visual color of meat surface. The color parameters (*L*,* a*, and b*) are suggested as the indicator of PSE (pale, soft, exudative) and/or DFD (dark, firm, dry) especially in poultry. In this study, αTPN effected the color parameters of fresh chicken meat. At day 14, higher concentration of αTPN (MIC-2 and MIC-4) significantly increased the pH and L* values. However, decreased a* values, increased b* and C* values, and a significant decrease in WHC was detected in meat containing a higher level of αTPN. It has been reported that if the broiler meat is very dark, pH will be high and if the meat is very light, it will have a low pH [63]; this was not witnessed in our result for αTPN-treated meat. It is known that muscles at pH ≤ 6.0 undergo greater protein denaturation and lead to an increase in light scattering and opaqueness properties of the meat [63]. The decrease of a* value during storage is due to the accumulation of MetMb pigment [64]. In general, it has been reported that a* values decreased with increasing storage period in the absence of oxygen in the package, while at 2 °C and in vacuum or MAP storage a* values can increase [64]. An increase in the water content of muscles leads to improve the quality and economical value of meat due to enhancing the tenderness, juiciness, firmness, and appearance [63]. It has been known that changes in meat pH can affect the WHC and meat quality; hence, a decrease in meat pH can lead to decreased WHC of muscle proteins [65]. In contrast, in our study, the increase in pH was observed with decreased WHC in meat treated with higher levels of αTPN. Myoglobin is commonly found in three forms: MetMb, DeoMb, and OxyMb, and the relative proportions of these determine the color of fresh meat [66]. In our study, the presence of αTPN decreased MetMb and DeoMb and increased OxyMb in chicken meat. Some studies demonstrate that natural preservatives can reduce oxidation of meat color and retard color loss by increasing the a* values and delaying MetMb formation [67]. The increase in fresh meat lightness is attributed to the increased auto-oxidation of OxyMb and the formation of reactive oxygen species [68].

### 4.3. Effect of αTPN on the TBARS Values of Chicken Meat

In this study, the meat containing αTPN (MIC-4) showed a pronounced effect against lipid oxidation by keeping TBARS scores lower than 2 mg MDA/kg in vacuum packaged ground chicken meat stored at 4 °C for 14 days. This could be attributed to the strong potential antioxidant activity of this BAC in inhibiting the formation of secondary products of lipid oxidation that may contribute to the off flavor in stored meat products. It has been shown that using ferric reducing antioxidant power (FRAP) and DPPH assays indicated that the αTPN possesses a strong antioxidant activity; this antioxidant activity is less compared to other oxygenated monoterpene BACs such as thymol and carvacrol. On the other hand, using the oxygen radical absorbance capacity (ORAC) assay, the αTPN (2.72 μmol Trolox equiv./μmol) could be compared to commercial antioxidants [42,60]. The protective effect of BACs (linalool and carvacrol) in chicken meat was also examined in our previous study, wherein both linalool and carvacrol showed great activity in reducing TBARS values and were active in protecting the color changes compared to the control group [36,37]. In a study by Bicas et al. [42] revealed that a range of 181–588 μM αTPN acts as a natural preservative with an antioxidant potential similar to BHA (butylated hydroxyanisole). Thus, αTPN attracts the interest for further research that can culminate in its use as a functional additive in food. To the best of our knowledge, no study has previously been conducted on the preservative potential of αTPN in controlling TBARS in chicken meat.

### 4.4. Effect of αTPN on the Microbiological Properties of Chicken Meat

Microbial spoilage has a huge effect on meat quality. In the current study, as the concentration of αTPN increased, the surviving count of AMCs decreased. It has been reported that that the AMCs in processed various cuts products of poultry (hamburgers, sausages) were approximately 7 log CFU/g and higher compared to fresh cuts (thighs, wings) with approximately 5.7 log CFU/g [69]. This could be due to an increase in the surface area of meat in contact with surfaces and air to increase the possibility of contamination. Additionally, 7 logs CFU/g have been used in some studies to define the spoilage for the criterion of microbiological acceptability of meat [70,71]. Zengin and Baysal [60] determined the MIC values of αTPN, which was 0.6% for *E. coli* O157:H7, *S. liquefaciens*, *C. divergens*, and *L. innocua*, while 0.7% of αTPN is needed to inhibit *S.* *aureus* and *S.* Typhimurium. In vitro study on the antimicrobial activity of αTPN reported that due to the presence of OH, this BAC interacts with intracellular components and causes the change in the permeability of the outer membrane and changes in the function of the cell membrane, leading to the leakage of intracellular materials. The EOs that are rich in αTPN have been used widely in folk medicine for aromatherapy due to their anti-spasmodic, antinociceptive, and immunostimulant properties. Li et al. [62] used transmission electron microscopy (TEM) and found that morphostructural alterations in *E. coli* induced MIC levels of αTPN and exhibited decreased cell size and irregular cell shape, cell wall and cell membrane were ruptured, plasmolysis occurred, nucleus cytoplasm was reduced, and nuclear area gathered aside. In this study, in control meat (without inoculation), no *L. monocytogenes* and *S.* Typhimurium were found at the beginning of storage. This demonstrates these pathogens were likely not initially present in the meat used. Park et al. [72] noticed that αTPN has strong antibacterial activities against *S. enteritidis* and *S.* *aureus*, in which the MIC and MBC values were 1.56, and 3.13 μL/mL, respectively. They also found that linalool and αTPN also exhibited strong antimicrobial activity against periodontopathic and cariogenic bacteria. They suggested that the concentration of BACs (linalool and αTPN) should be kept below 0.4 mg/mL for use as components of toothpaste or gargling solution. In another study, Park et al. [73] studied the antifungal activity of terpenes with the concentrations of 0.09 and 0.2 mg/mL for citral, 0.4 mg/mL for eugenol, 0.4 mg/mL for nerolidol, and 1 mg/mL for αTPN. They found αTPN had the lowest antifungal activity among all the evaluated terpenes. Moreover, it is known that the presence of free hydroxyl group is essential for antimicrobial activity of BACs and that compound could act as a protonophore which is applicable for αTPN [74]. Additionally, it has been reported that at the aerobic condition the meat product should have an initial load of *Pseudomonas* spp. fewer than 100 CFU/g to achieve an ideal shelf life and sensory demand [21]. On the basis of the effect of αTPN on AMC and *P. lundessis* in meat, the MIC-1 produced less than 7 log CFU/g. This indicates that the meat shelf life was increased by up to 2 weeks of storage time. However, further accurate presence/absence detection test might be beneficial to determine the safety of meat regarding the pathogens *L. monocytogenes* and *S.* Typhimurium.

### 4.5. Effect of αTPN on the Smell Detection by Electronic-Nose in Chicken Meat

In this study, E-nose showed a visible distinguishment between untreated and treated meat on the basis of the type of BAC and storage time. It is the case that the single compound that is primarily responsible for aroma of meat has not been identified yet, while an aroma profile (fingerprint) that is a combination of volatile compounds may be used as an indicator of spoilage or to differentiate between types of meat [75]. Generally, chicken meat becomes spoiled in a short period of time, and despite the storage at 4 °C in refrigerator condition, the shelf life of chicken meat is very short (almost 3 days) [76]. In the current study, after opening the bags that contained treated meat, intense odor of eucalyptus globulus, pine oil, and marjoram were noticed, which could be pleasant to some consumers. Alongside higher pH and L* values, a reduction in TBARS and in the bacterial count was noticed with αTPN (MIC-2 and MIC-4), indicating that the E-nose instrument can classify the chicken meat as either fresh or spoiled with rancid flavor. However, the effect of αTPN as a natural BAC on sensory quality (flavor, texture) of meat (either raw or ready to eat meat) and detecting its minimum acceptable concentration in association with the lipid oxidation and microbiological characteristics still needs to be established.

## 5. Conclusions

There is an increasing trend in using natural preservatives such as bioactive compounds in food preservation. To the best of our knowledge, no researcher has used α-terpineol in fresh chicken meat preservation. In the current study, the BAC (αTPN) exhibited a great antibacterial activity in the in vitro experiment. In the chicken meat experiment, the different concentrations of αTPN were able to alter the physicochemical attributes, showing a reduction in lipid oxidation, and altered the meat pigments of chicken meat during refrigerated conditions. Additionally, the E-nose differentiated chicken meat groups by detecting the different concentrations of αTPN; however, future experiments need to be conducted to use E-nose for classifying the meat treated with BACs as either fresh or spoiled with rancid flavor. Furthermore, the αTPN at a particularly high level (MIC-4) showed strong antimicrobial activity against aerobic mesophilic counts, *P. lundessis, L. monocytogenes,* and *S.* Typhimurium. The present work suggests that low concertation of αTPN has great potential to improve the quality of fresh chicken meat. Nevertheless, further studies are required to consider the consumer’s perception towards the sensorial attributes of ready-to-eat meat treated with this BAC.

## Figures and Tables

**Table 1:** Antibacterial activity using filter paper disc diffusion estimated by inhibition zone of different BACs against *P.* *lundensis*, *E.* *coli* O157:H7, *S.* *aureus*, *L.* *monocytogenes*, *S.* Typhimurium, and *B.* *cereus*.

Bacterial Strains	Storage Time (h)	Bioactive Compounds								
		p-Cymene	Linalool	Camphor	Piperine	γ-Terpinene	α-Terpineol	α-Pinene	1,8-Cineole	Carvacrol
*Pseudomonas* *lundensis*	24	NI	1.10 ± 0.06	NI	NI	NI	1.83 ± 0.01	0.93 ± 0.06	NI	5.19 ± 0.02
	48	NI	1.10 ± 0.15	NI	NI	NI	1.41 ± 0.38	0.84 ± 0.18	NI	5.16 ± 0.17
	72	NI	1.03 ± 0.00	NI	NI	NI	0.94 ± 0.01	0.89 ± 0.00	NI	5.17 ± 0.06
*Escherichia coli*	24	NI	7.12 ± 0.07	1.02 ± 0.00	NI	NI	3.02 ± 0.15	0.77 ± 0.78	2.50 ± 0.60	20.14 ± 0.73
	48	NI	7.42 ± 0.66	1.20 ± 0.00	NI	NI	2.84 ± 0.03	0.53 ± 0.53	1.75 ± 0.22	16.94 ± 0.66
	72	NI	6.66 ± 0.30	1.04 ± 0.00	NI	NI	2.24 ± 0.57	0.63 ± 0.63	2.02 ± 0.18	16.76 ± 0.92
*Staphylococcus aureus*	24	0.88 ± 0.89	4.01 ± 0.23	NI	NI	1.02 ± 1.03	2.83 ± 0.01	2.62 ± 0.77	NI	16.70 ± 0.29
	48	0.78 ± 0.78	3.65 ± 0.23	NI	NI	0.73 ± 0.74	2.42 ± 0.72	1.61 ± 0.26	NI	16.20 ± 0.60
	72	0.65 ± 0.65	3.38 ± 0.25	NI	NI	0.64 ± 0.64	1.76 ± 0.25	1.44 ± 0.20	NI	15.86 ± 0.09
*Listeria monocytogenes*	24	NI	3.43 ± 0.15	NI	NI	NI	1.86 ± 0.76	NI	NI	17.27 ± 1.00
	48	NI	3.10 ± 0.02	NI	NI	NI	1.13 ± 0.03	NI	NI	17.01 ± 1.57
	72	NI	2.83 ± 0.36	NI	NI	NI	1.31 ± 0.40	NI	NI	17.07 ± 0.99
*Salmonella* Typhimurium	24	NI	5.19 ± 0.37	NI	NI	1.06 ± 0.06	2.33 ± 0.04	1.22 ± 0.02	1.25 ± 0.25	15.15 ± 0.27
	48	NI	4.86 ± 0.91	NI	NI	1.32 ± 0.02	2.09 ± 0.46	0.87 ± 0.04	1.10 ± 0.40	15.45 ± 0.34
	72	NI	5.22 ± 0.40	NI	NI	0.80 ± 0.30	1.99 ± 0.38	0.83 ± 0.15	1.12 ± 0.45	15.80 ± 0.24
*Bacillus cereus*	24	NI	4.30 ± 0.52	NI	NI	NI	2.52 ± 0.60	NI	NI	17.60 ± 0.39
	48	NI	3.84 ± 0.61	NI	NI	NI	2.07 ± 1.10	NI	NI	14.14 ± 0.12
	72	NI	3.55 ± 0.82	NI	NI	NI	1.50 ± 1.02	NI	NI	15.35 ± 0.09

NI: no inhibition, αTPN: α-terpineol. Thickness of inhibition zone was calculated in (mm ± SD).

**Table 2:** Minimum inhibitory concentration (MIC μL/mL) of various BACs against *P. lundensis,* *E.* *coli* O157:H7, *S.* *aureus*, *L.* *monocytogenes*, *S.* Typhimurium, and *B.* *cereus*.

Bacterial Strains	Ethanol	Bioactive Compounds								
		p-Cymene	Linalool	Camphor	Piperine	γ-Terpinene	αTPN	α-Pinene	1,8-Cineole	Carvacrol
*Pseudomonas* *lundensis*	NI	0.125	0.125	0.5	0.5	0.5	0.125	1	0.5	0.25
*Escherichia coli*	NI	0.5	0.125	0.5	0.5	1	0.125	1	0.5	0.063
*Staphylococcus aureus*	NI	0.5	0.125	0.5	0.5	1	0.25	1	0.125	0.063
*Listeria monocytogenes*	NI	0.5	0.125	0.5	0.5	0.5	0.25	0.5	0.25	0.125
*Salmonella* Typhimurium	NI	0.5	0.125	0.5	0.5	0.5	0.25	0.5	0.5	0.25
*Bacillus cereus*	NI	0.5	0.25	0.5	0.5	1	0.25	0.5	0.5	0.125

NI: No inhibition, αTPN: α-terpineol.

**Table 3:** The influence of different concentrations of αTPN on pH, color values, and WHC of fresh chicken meat stored up to 14 days at 4 °C.

Parameters	Storage Time (d)	Treatments			
		No-BAC	αTPN-MIC-1	αTPN-MIC-2	αTPN-MIC-4
pH	0	6.02 ± 0.02 ^aA^	6.02 ± 0.02 ^abA^	6.04 ± 0.01 ^aAB^	6.09 ± 0.03 ^aB^
	3	6.00 ± 0.02 ^aA^	6.01 ± 0.02 ^aAB^	6.04 ± 0.00 ^aB^	6.11 ± 0.01 ^aC^
	7	6.00 ± 0.01 ^aA^	6.02 ± 0.01 ^abA^	6.04 ± 0.02 ^aA^	6.12 ± 0.00 ^aB^
	10	6.01 ± 0.01 ^aA^	6.03 ± 0.01 ^abAB^	6.05 ± 0.01 ^abB^	6.11 ± 0.02 ^aB^
	14	6.01 ± 0.01 ^aA^	6.04 ± 0.01 ^bB^	6.06 ± 0.01 ^bB^	6.12 ± 0.01 ^aC^
L*	0	46.75 ± 1.01 ^aA^	49.82 ± 0.59 ^aB^	50.44 ± 0.67 ^aB^	55.33 ± 1.24 ^aC^
	3	47.69 ± 0.69 ^aA^	49.73 ± 1.75 ^aA^	52.25 ± 1.24 ^abB^	58.56 ± 0.91 ^bC^
	7	47.23 ± 1.39 ^aA^	49.02 ± 0.96 ^aA^	51.95 ± 0.57 ^abB^	58.66 ± 1.64 ^bC^
	10	47.46 ± 0.66 ^aA^	49.32 ± 0.40 ^aB^	52.58 ± 1.32 ^bC^	59.40 ± 1.00 ^bD^
	14	48.30 ± 1.32 ^aA^	50.02 ± 0.66 ^aA^	52.47 ± 1.28 ^bB^	59.00 ± 0.79 ^bC^
a*	0	1.41 ± 0.46 ^aA^	1.36 ± 0.38 ^aA^	1.66 ± 0.22 ^aA^	1.75 ± 0.46 ^aA^
	3	1.66 ± 0.74 ^aA^	1.27 ± 9.78 ^aA^	1.67 ± 0.39 ^aA^	1.87 ± 0.74 ^aA^
	7	1.69 ± 0.74 ^aA^	1.26 ± 0.039 ^aA^	1.61 ± 0.44 ^aA^	1.79 ± 0.57 ^aA^
	10	1.80 ± 0.18 ^aA^	1.36 ± 0.18 ^aA^	1.52 ± 0.25 ^aA^	1.72 ± 0.21 ^aA^
	14	1.87 ± 0.44 ^aA^	1.37 ± 0.64 ^aA^	1.52 ± 0.30 ^aA^	1.72 ± 0.35 ^aA^
b*	0	12.78 ± 0.72 ^aA^	12.59 ± 1.16 ^aA^	13.08 ± 0.68 ^aA^	15.29 ± 1.56 ^bB^
	3	12.05 ± 2.11 ^aA^	12.58 ± 1.14 ^aAB^	14.79 ± 1.16 ^abBC^	16.90 ± 0.86 ^bC^
	7	11.34 ± 1.12 ^aA^	12.42 ± 0.30 ^aA^	14.71 ± 0.94 ^abB^	16.98 ± 1.52 ^bC^
	10	11.19 ± 0.81 ^aA^	12.26 ± 0.51 ^aA^	14.51 ± 0.46 ^abB^	16.82 ± 1.35 ^bC^
	14	11.66 ± 0.28 ^aA^	12.55 ± 0.53 ^aA^	15.00 ± 1.18 ^bB^	16.62 ± 1.08 ^bC^
C*	0	12.87 ± 0.68 ^aA^	12.67 ± 1.18 ^aA^	13.18 ± 0.68 ^aA^	15.40 ± 1.57 ^aB^
	3	12.18 ± 2.13 ^aA^	12.66 ± 1.19 ^aAB^	14.89 ± 1.14 ^abBC^	17.02 ± 0.82 ^aC^
	7	11.49 ± 1.06 ^aA^	12.48 ± 0.31 ^aA^	14.80 ± 0.93 ^abB^	17.09 ± 1.47 ^aC^
	10	11.33 ± 0.85 ^aA^	12.34 ± 0.50 ^aA^	14.59 ± 0.45 ^abB^	16.91 ± 1.34 ^aC^
	14	11.81 ± 0.33 ^aA^	12.63 ± 0.57 ^aA^	15.08 ± 1.16 ^bB^	16.71 ± 1.06 ^aC^
h*	0	1.46 ± 0.04 ^aA^	1.46 ± 0.02 ^aA^	1.44 ± 0.02 ^aA^	1.46 ± 0.03 ^aA^
	3	1.43 ± 0.05 ^aA^	1.47 ± 0.05 ^aA^	1.46 ± 0.03 ^aA^	1.46 ± 0.05 ^aA^
	7	1.42 ± 0.07 ^aA^	1.47 ± 0.03 ^aA^	1.46 ± 0.03 ^aA^	1.46 ± 0.04 ^aA^
	10	1.41 ± 0.03 ^aA^	1.46 ± 0.02 ^aB^	1.47 ± 0.02 ^aB^	1.47 ± 0.02 ^aB^
	14	1.41 ± 0.03 ^aA^	1.46 ± 0.05 ^aB^	1.47 ± 0.02 ^aB^	1.47 ± 0.02 ^aB^
WHC (%)	0	2.02 ± 0.74 ^aA^	1.96 ± 0.48 ^aA^	1.81 ± 0.12 ^aA^	2.45 ± 0.39 ^aA^
	3	1.74 ± 0.26 ^aA^	2.01 ± 0.20 ^aA^	1.88 ± 0.15 ^aA^	2.70 ± 0.22 ^abB^
	7	1.80 ± 0.13 ^aA^	2.44 ± 0.43 ^aAB^	3.07 ± 0.55 ^bB^	2.89 ± 0.17 ^abB^
	10	1.90 ± 0.19 ^aA^	2.10 ± 0.17 ^aAB^	2.92 ± 0.37 ^bBC^	3.56 ± 0.51 ^bC^
	14	1.96 ± 0.50 ^aA^	2.58 ± 0.40 ^aAB^	2.77 ± 0.06 ^bAB^	3.46 ± 0.29 ^bB^

^a,b^—letters in the same column with different superscript are significantly different regarding the days of storage; ^A,B,C^—letters in the same row with different superscript are significantly different regarding the concentrations of BACs (*p* < 0.05). Color values: *L*^∗^, lightness; *a*^∗^, redness; *b*^∗^, yellowness; C*, chroma; h*, hue angle. WHC, water holding capacity.

## Data Availability

Not applicable.

## Appendix A

**Table A1:** Concentration and dilution of αTPN applied in meat.

Total weight	100	g						
MIC1:	2.5	g αTPN in	1000	g final mixture		let 1 mL αTPN is 1 g αTPN		
if	2.5	g αTPN in	1000	g final mixture				
then	0.25	g αTPN in	100	g final mixture				
				αTPN	0.25	g		
			+	ethanol	1.25	g		=5× weight of αTPN
				αTPN + ethanol	1.5	g		
			+	DW	3.45	g		
				αTPN + DW + ethanol	5	g	5	% of meat
			+	meat	95	g	95	% of total weight
					100	g		total weight
The concentration of αTPN in MIC-2 were ×2, and in MIC-4 were ×4								
